# Alterations in ERBB2 and BRCA and microsatellite instability as new personalized treatment options in small bowel carcinoma

**DOI:** 10.1186/s12876-019-0942-z

**Published:** 2019-02-04

**Authors:** Alexander Quaas, Carina Heydt, Dirk Waldschmidt, Hakan Alakus, Thomas Zander, Tobias Goeser, Philipp Kasper, Christiane Bruns, Anna Brunn, Wilfried Roth, Nils Hartmann, Anne Bunck, Matthias Schmidt, Reinhard Buettner, Sabine Merkelbach-Bruse

**Affiliations:** 1 Institute of Pathology, University of Cologne, Cologne, Germany; 2 Department of Visceral Surgery, University of Cologne, Cologne, Germany; 3 Department of Oncology and Hematology, University of Cologne, Cologne, Germany; 4 Department of Hepato- and Gastroenterology, University of Cologne, Cologne, Germany; 5 Institute of Neuropathology, University of Cologne, Cologne, Germany; 6 Institute of Pathology, University of Mainz, Mainz, Germany; 7 Department of Radiology, University of Cologne, Cologne, Germany; 8 Department of Nuclear Medicine, University of Cologne, Cologne, Germany

**Keywords:** Small bowel adenocarcinoma, *BRCA* mutation, PARP-inhibition, *ERBB2* mutation, Microsatellite-instability

## Abstract

**Background:**

Carcinomas of the small bowel are rare tumors usually with dismal prognosis. Most recently, some potentially treatable molecular alterations were described. We emphasize the growing evidence of individualized treatment options in small bowel carcinoma.

**Methods:**

We performed a DNA- based multi-gene panel using ultra-deep sequencing analysis (including 14 genes with up to 452 amplicons in total; *KRAS, NRAS, HRAS, BRAF, DDR2, ERBB2, KEAP1, NFE2L2, PIK3CA, PTEN, RHOA*, *BRCA1, BRCA2 and TP53)* as well as an RNA-based gene fusion panel including *ALK, BRAF, FGFR1, FGFR2, FGFR3, MET, NRG1, NTRK1, NTRK2, NTRK3, RET* and *ROS1* on eleven formalin fixed and paraffin embedded small bowel carcinomas. Additionally, mismatch-repair-deficiency was analyzed by checking the microsatellite status using the five different mononucleotide markers *BAT25, BAT26, NR-21, NR-22* and *NR-27* and loss of mismatch repair proteins using four different markers (*MLH1, MSH6, MSH2, PMS2*).

**Results:**

In five out of eleven small bowel carcinomas we found potentially treatable genetic alterations. Three patients demonstrated pathogenic (class 5) *BRCA1* or *BRCA2* mutations – one germline-related in a mixed neuroendocrine-non neuroendocrine neoplasm (MiNEN). Two additional patients revealed an activating *ERBB2* mutation or *PIK3CA* mutation.

Furthermore two tumors were highly microsatellite-instable (MSI-high), in one case associated to Lynch-syndrome. We did not find any gene fusions.

**Conclusion:**

Our results underscore, in particular, the relevance of potentially treatable molecular alterations (like *ERBB2, BRCA* and MSI) in small bowel carcinomas. Further studies are needed to proof the efficacy of these targeted therapies in small bowel carcinomas.

## Background

Small intestinal carcinomas account for about 3% of all gastro-intestinal tract tumors [1]. Recent epidemiological data were reported from a Netherland registry showing a 0.7 / 100,000 incidence [2]. Small bowel carcinoma has an annual incidence of about 6 cases per million and is therefore much rarer than colo-rectal carcinomas, which have an incidence of about 420 cases per million inhabitants in the same period (1995–2002) [3–5].

The most common tumor location is the duodenum, followed by jejunum and ileum. According to a French study men and women are equally effected. The mean age at occurrence is in their middle to late sixties [6].

The main cause of small bowel carcinoma is unknown. Predisposing factors can be chronic inflammatory bowel syndromes, celiac disease or Lynch-syndrome [3]. In general, the prognosis is worse than for colon carcinoma [7].

According to one publication distal tumor location (ileum) is one predictor for poor survival [8]. On the other hand a recent large study reported that duodenum localisation is a negative predictor of survival after resection of SBA [9].

Thus, there is a strong need for a more effective and personalized systemic treatment option in small bowel carcinoma due to limited effects of standard chemotherapy-based treatments in a metastatic setting.

Seven studies in the past (in years: 1997–2014 analyzed between 15 and 89 tumors each) found 5–35% MSI-small bowel carcinomas. More recently Schrock, A et al. [10] described molecular alterations in 317 small bowel carcinomas as well as Hänninen et al. [1] in additional 160 SBACs. Microsatellite-instability (MSI) was found in 7.6% [10] and 14.2% [1], respectively.

The main molecular mutations considering both publications include (up to): *TP53* (48.0%), *KRAS* (53.6%), *APC* (26.8%), *CDKN2A* (14.5%), *SMAD4* (17.4%), *SOX9* (12.0%) and *BRAF* (9.1, 10% of these *BRAF* mutated cases in the study of Schrock showed the common p.V600E mutation whereas Hänninen did not find any *BRAF* V600E mutations) as well as *ERBB2* mutations in 8.2%. [1, 10].

Furthermore, Hänninen et al. described novel candidate driver genes like *ACVR1B, BRCA2,* and *SMARCA4*. Copy number gains were observed mainly in *KRAS* (18.9%), *BRAF* (17.9%) and *PIK3CA* (15.3%). Nearly 10% of the Finnish patient cohort suffered from celiac disease. This group revealed a higher amount of MSI tumors [1].

In our study eleven small bowel carcinomas were analyzed focusing on individualized treatment options and on DNA-repair deficiency including *BRCA* mutations.

## Methods

Eleven small bowel carcinomas were selected from the registry of the Institute of Pathology of the University Hospital Cologne, Germany. We identified these cases over a time-frame of six years. We considered primary small bowel tumors (no metastasis) with existing paraffin material for further molecular analyses. Ten of these patients had adenocarcinomas, one a mixed neuroendocrine-non neuroendocrine neoplasm (MiNEN) of the small bowel. All samples were routinely formalin-fixed and paraffin embedded (FFPE) according to local practice.

### Parallel sequencing

All tumors were analyzed for a panel of 14 different genes including *RAS (K, N, H-RAS), DDR2, BRAF, ERBB2, KEAP1, PIK3CA, NFE2L2, PTEN, TP53, RHOA, BRCA1* and *BRCA2* resulting in a total of 452 amplicons. The gene panel includes also 14 different microsatellite regions [11, 12].

Areas of carcinoma were marked on H&E-stained slides by an experienced pathologist and DNA was extracted by manual macro-dissection – details are summarized in [24].

### Classification of *BRCA* variants

According to the established IARC classification each *BRCA* variant was classified [13] including class 1 variants (not pathogenic) via class 2, class 3, class 4 to class 5 variants (definitively pathogenic). For assessment of variants, the following databases were used:

ARUP BRCA mutation database: http://arup.utah.edu/database/BRCA/

ClinVar database: http://www.ncbi.nlm.nih.gov/clinvar/

Universal mutation database BRCA share: http://www.umd.be/

Leiden Open Variation Database: http://chromium.lovd.nl/LOVD2/

Variants that are not listed in the above mentioned databases were classified according to the ENIGMA criteria (https://enigmaconsortium.org/).

### RNA-based fusion panel analysis

Six sections of 10 μm thickness were deparaffinized and the tumor areas were macrodissected from unstained slides using a marked hematoxylin-eosin (H&E) stained slide as a reference. Total nucleic acid was extracted with the Maxwell RSC RNA FFPE Kit on the Maxwell RSC (Promega) according to manufacturer’s instruction, only the DNase solution during the digestion step was replaced by 50 μl water.

Total nucleic acid extracts were quantified with the Qubit RNA BR Assay Kit (Thermo Fisher Scientific) on the Qubit 2.0 Fluorometer (Thermo Fisher Scientific). For the detection of gene fusions the Archer FusionPlex CTL panel (Archerdx, Boulder, CO, USA) was used according to manufacturer’s instructions. In brief, 35–200 ng tNA were target-enriched and prepared cDNA libraries were sequenced on a MiSeq (Illumina). For data analysis and fusion detection the Archer Analysis Software (Archerdx) was used. Strong and weak evidence fusions were evaluated whilst taking into account the read statistics and assay targets.

### Immunohistochemical analysis of mismatch-repair deficiency (MMR)

All tumors were stained for *MLH1, MSH2, MSH6, PMS2* (using *MLH1* (Clone:M1 Ventana), *MSH6* (Clone44, Ventana), *PMS2* (Clone:EPR3947, Cell Marque), *MSH2* (Clone:G219–1129, Cell Marque)) on Ventana Benchmark stainers. 3,3′-Diaminobenzidine (DAB) was used as a chromogen and hematoxylin as a counterstain.

## Results

We analyzed formalin-fixed and paraffin-embedded tumor material of eleven patients in total (for patients’ characteristics see Table 1). In about half of our cohort we found potentially treatable genomic alterations in the genes *BRCA*, *ERBB2* and *PIK3CA* as well as microsatellite instability (see Table 2).

**Table 1:** Patients’ characteristics

N°	Age range	Localisation	TNM stage
1	55–60	Jejunum	T4N1M0
2	70–75	Duodenum	T3N1M0
3	70–75	Ileum	T3N1M1
4	45–50	Duodenum	T2N0M0
5	65–70	Jejunum	T2N1M0
6	45–50	Duodenum	T4N1M0
7	55–60	Jejunum	T2N0M0
8	45–50	Ileum	T4N0M1
9	55–60	Jejunum	T4N0M1
10	60–65	Ileum	T4N1M1
11	50–55	Ileum	T4N0M0

**Table 2:** Molecular alterations in small bowel carcinomas in our cohort

N°	BRCA	TP53	ERBB2	KRAS	MSI	PIK3CA	KEAP1
1	BRCA2 p.N986Ifs*5	p.R273C	wt	wt	MSI	wt	p.Y490C
2	wt	p.C135Y	wt	p.G12D	n.a.	wt	wt
3	BRCA1 p.V1234Qfs*8	p.C275Y	wt	wt	MSS	wt	wt
4	BRCA1 p.E1540*	wt	wt	wt	MSS	wt	p.R320L
5	wt	p.G245S	p.T862A	wt	MSS	wt	wt
6	wt	wt	wt	p.G12 V	MSS	p.E545G	wt
7	BRCA1 p.T77 M BRCA2 p.R1512C	wt	wt	p.A146T	MSI Lynch-syndrome	p.E542K	p.R202H
8	wt	p.R175H	wt	p.G12 V	MSS	wt	wt
9	wt	wt.	wt.	p.G12C	MSS	wt	wt
10	wt	p.R248Q	wt.	wt	MSS	p.E542K	wt
11	wt	wt	wt	wt	MSS	wt	wt

*wt* wildtype, *MSI* microsatellite instable, *MSS* microsatellite stable, *n.a.* not analyzable

## Molecular alterations

### Parallel sequencing

Mutational analysis by parallel sequencing was feasible in all eleven tumors. In ten out of eleven tumors the microsatellite status could be determined using five different mononucleotide markers: *BAT25, BAT26, NR-21, NR-22* and *NR-27* [14].

Four out of eleven tumors revealed a *BRCA* mutation (two cases with *BRCA1* mutations, one case with a *BRCA2* mutation and an additional case with known Lynch-syndrome showing co-occurrence of *BRCA1* and *BRCA2* mutations). According to different databases (UMD, ARUP, ClinVar) all *BRCA* mutations were classified as pathogenic (class 5) except the Lynch-syndrome associated *BRCA*-mutations which were classified as class 3 non-pathogenic *BRCA* mutations. One patient (patient 3) harbors a germline-related *BRCA1* mutation and developed a mixed neuroendocrine-non neuroendocrine neoplasm (MiNEN) in his ileum. The patient was microsatellite stable (MSS) and showed a mutation in *TP53* (p.C275Y) leading to a non-functional protein. The mutational hot spots of all other analyzed genes in this case were wild type.

The other *BRCA* mutated carcinomas also presented with co-occuring mutations:

Patient 1 showed a *KEAP1* mutation in exon 4: c.1469A > G p.Y490C (allele frequency of 38.6%), a *TP53* mutation in exon 8: c.817C > T p.R273C (allele frequency of 36.5%) and wild type sequences in the mutational hot-spots of all other genes tested. For the tumor of this patient we confirmed microsatellite instability (MSI) using the five different markers as describe above and confirmed the loss of DNA repair proteins using immunohistochemistry.

Patient 4 showed a *KEAP1* mutation in exon 3: c.959_960GG > TT p.R320L (allele frequency of 29.5%) and wild type sequences in the mutational hot-spots of all other genes tested. This tumor was microsatellite stable.

Altogether, 6/11 tumors (55%) showed a *TP53* mutation, 5/11 tumors (45%) showed a *KRAS* mutation, 4/11 tumors (36%) a *BRCA* mutation, 3/11 tumors (27%) a *PIK3CA* mutation, 3/11 tumors (27%) a *KEAP1* mutation, 2/10 tumors (20% were MSI and 1/11 carcinomas (9%) a mutation in *ERBB2.* (Table 2).

### RNA-based fusion panel analysis

Due to limited availability of tissue, only six out of eleven tumors were analyzable by RNA sequencing. In none of these tumors a gene fusion was detected with the Archer FusionPlex CTL panel.

## Discussion

In this study we were able to confirm the results of Hänninen et al., who described for the first time pathogenic and therapeutically relevant *BRCA2* mutations in their analyses of 106 SBAC. Additionally, we found two patients with pathogenic *BRCA1* mutations, and one of them turned out to be germline related (patient 3). In patient 4 the *BRCA1* mutation was a point mutation with a low allele frequency of 5.5% leading to a truncated protein (p.E1540*) and described as pathogenic in the ARUP and ClinVar databases. In a third patient a somatic truncating *BRCA2* mutation in exon 11 with an allele frequency of 38.0% was detected (patient 1, p.N986Ifs*5). According the ENIGMA criteria this truncating mutation is likely pathogenic (class 4). The tumor of a fourth patient with known Lynch-syndrome revealed a *BRCA1* as well as a *BRCA2* mutation both classified as class 3 mutations and therefore probably not therapeutically important. Both mutations are presumably due to the microsatellite-instability-related higher mutational burden.

DNA repair is essential to maintain DNA integrity – *BRCA1* as well as *BRCA2* deficient cells show a high degree of chromosomal instability, increasing the risk of malignant transformation [15–18]. Ovarian carcinomas with a somatic *BRCA* mutation are likely to respond equally well to therapies that include PARP inhibitors as those with germline related *BRCA-*mutations [9, 16, 19–23].

In the recent study by Hänninen et al. *BRCA* mutation were detected for the first time in 7% of 106 patients [1] In our study *BRCA* mutations were detected with an even higher percentage of 36% (Table 3). However, results could be hampered by the small sample size.

**Table 3:** Major molecular alterations in small bowel adenocarcinoma described by others in comparison to our study

References	No. of patients	TP53 (%)	KRAS (%)	ERBB2 (%)	MSi (%)	BRCA (%)
Hänninen et al. (2018) [1]	106	48	47	14	14.1	5
Schrock et al. (2017) [10]	317	51	53.6	8.2	7.6	n.a
Laforest et al. (2014) [26]	83	41	43	12	21	n.a
Overman et al. (2012) [7]	54	–	–	–	–	n.a
Planck et al. (2003) [31]	89	–	–	–	–	n.a
Quaas et al. (2018) [24]	11	55	45	9	20	36

We also confirmed the importance of pathogenic *BRCA* mutations. In the case of the germline-related *BRCA1* mutated MiNEN we could successfully proof the efficacy of a combination of platin-based chemotherapy and the PARP inhibitor olaparib; more than two years after his initial diagnosis of a diffuse metastasized MiNEN (cerebral and different liver metastases) the patient is in a general good condition still without metastases [24].

In addition, first indications are reported that *BRCA*-mutated ovarian cancers respond well to immuno-checkpoint inhibitors. This is probably due to the higher mutation burden of these tumors compared to *BRCA* non-mutated ovarian carcinoma [25]. It remains to be shown whether *BRCA* mutated small bowel adenocarcinoma also benefit from immune-checkpoint inhibition as a second option after PARP inhibition.

*ERBB2* is a well-known tyrosine kinase and belongs to the *ERBB*-family (*ERBB1–4*). *ERBB2* amplification is especially important in breast- and gastric carcinomas and is therapeutically targetable using e.g. the tyrosine-kinase inhibitor trastuzumab. Few publications describe the importance of *ERBB2* mutations in small bowel carcinomas [1, 10, 26]. Recent studies by Schrock et al. and Hänninen et al. found comparable results to our study with 8.2 and14% of activating *ERBB2* mutations in their patient population [1, 10] In our study we detected in 9% of patient samples an *ERBB2* mutation (Table 3). The majority of the *ERBB2* mutations clustered into four known hotspots (L755S, was found exclusively in MSI tumors), S310F/Y, R678Q, and V842I). Concurrent hotspot mutations were reported. In concordance with the results mentioned above we could also detect potentially treatment sensitive *ERBB2* mutations in our cohort (9%). Our *ERBB2* mutated tumor revealed a activating exon 21 mutation (c.2584 A > G p.T862A) which is sensitive to inhibition by neratinib and lapatinib [26].

According to previous studies microsatellite instability (MSI) occurs in a significant number of cases (5–35%). MSI can be germline-related (Lynch-syndrome like in one patient in our cohort) or more frequently somatically induced by an epigenetic silencing of the MLH1 promotor.

Currently, the largest studies by Schrock et al. and Hänninen et al. found high-levels of MSI in SBCAs in 7.6 and 14.1%. We demonstrated a high-level MSI in two out of ten analyzable patients (20%) in our cohort (Table 3). There is growing evidence that microsatellite-instable tumors as well as tumors with a high tumor mutational burden respond well to checkpoint inhibitors and that microsatellite as well as tumor mutational burden status can predict therapy outcome [27].

Results from the keynote studies 158 and 164 confirmed the anti-tumor efficacy of the PD-1-inhibitor pembrolizumab in patients with microsatellite instable colon cancers. Pembrolizumab was approved by the FDA in 2017 for MSI high or mismatch-repair-deficient solid tumors irrespective of tumor origin [28].

The most often altered cancer pathway in SBAC is the PI3K/AKT-pathway. In line with this activating *PIK3CA* mutations were described in 16% of SBCA by Schrock eal. and were confirmed by us (27%). Tumors driven by an activated PI3K/AKT-pathway might benefit from treatment with a PIK3CA or MEK inhibitor. Currently, clinical trials are ongoing with targeted therapies for *PIK3CA* mutated tumors of different entities (https://clinicaltrials.gov, for example: NCT02389842, NCT02644122). These therapies are based on convincing preclinical and clinical studies [29, 30].

Beyond the mutations described above (*ERBB2*, *BRCA* and *PIK3CA*) the tumors we investigated revealed additional mutations including in the genes *KEAP1*, *KRAS* and *TP53* (compare Table 3). There is a growing evidence that *TP53* mutated tumors harbor a higher mutational burden and a higher chromosomal instability in comparison to *TP53* wild type tumors. Until now nothing is known about different treatment responses to e.g. checkpoint inhibition considering the *TP53* mutational status of the tumors. TP53 mutations were detected in our cohort in 55% of samples. This is also in concordance with Schrock et al. and Hänninen et al. Here, PIK3CA mutations were detected with 51 and 48% [1, 10] (Table 3).

Limitations of our study include the small number of cases analyzed. Nevertheless, we were able to demonstrate important and rarely described molecular alterations in SBAC (e.g. *BRCA* mutations) and could confirm findings of much larger studies (e.g. Schrock et al. and Hänninen et al.) in our small collective. We and other detected potentially targetable molecular alterations with similar percentages (e.g. microsatellite-instability or activating *ERBB2*-mutations) (Table 3) in SBAC. However, the percentage of BRCA mutations in our smaller sample cohort was higher than previously published. In the future, further clinical validations are needed regarding treatment response. Currently, treatment response against specific genomic alterations in SBACs was extrapolited from other tumor entities like colon or gastric carcinoma.

Parallel sequencing on RNA using the Archer FusionPlex CTL panel did not reveal any gene fusions.

## Conclusion

Our results underscore, in particular, the relevance of potentially treatable molecular alterations (like *ERBB2, BRCA* and MSI) in small bowel carcinomas. Further clinical studies are needed to proof the efficacy of these targeted therapies in small bowel carcinomas.

## Abbreviations

BRCA1: Breast cancer gene 1
FFPE: Formalin-fixed and paraffin embedded
H&E: Hematoxylin-eosin
HR: Homologous recombination
MiNEN: Mixed neuroendocrine-non neuroendocrine neoplasm
MMR: Mismatch repair deficiency
MSI: Microsatellite-instable
MSS: Microsatellite stable
PARP: Poly (ADP-Ribose)-Polymerase
SBAC: Small-bowel adenocarcinoma
SBCA: Small-bowel carcinoma
